# Acute promyelocyte leukemia arose from *CALR* 1 mutated post essential thrombocythemia- myelofibrosis with splanchnic vein thrombosis: A case report

**DOI:** 10.1016/j.lrr.2021.100243

**Published:** 2021-05-08

**Authors:** E Morsia, G Goteri, E Torre, KB Garvey, G Discepoli, A Tassoni, S Mancini, F Giantomassi, A Poloni, A Olivieri, S Rupoli

**Affiliations:** a Clinica Ematologica, Ospedali Riuniti di Ancona, Ancona, Italy; b Anatomia Patologica, Ospedali Riuniti di Ancona, Ancona, Italy; c Università Politecnica delle Marche, Ancona, Italy; d Laboratorio di Genetica Medica, Ospedali Riuniti di Ancona, Ancona, Italy; e Laboratorio Clinica di Ematologia, Università Politecnica delle Marche, Ancona, Italy

**Keywords:** Essential thrombocytemia, Myelofibrosis, Blast-phase myeloproliferative neoplasm, Splanchnic vein thrombosis, Acute promyelocyte leukemia

## Abstract

Major disease complications for patients with essential thrombocythemia (ET) include thrombosis and fibrotic or leukemic transformation. Calreticulin (CALR) mutation type 1 frequencies in ET are estimated between 7% and 11% and ET patients carrying *CALR* type 1 mutation are associated with lower risk of thrombosis but higher risk of myelofibrosis transformation compared to ET patients with *JAK2* mutation. Leukemic transformation rates at 20 years are estimated at less than 5% for ET and risk factors for leukemic transformation are advanced age, thrombosis history, leukocytosis, and anemia. Amongst the subtypes of blast phase myeloproliferative neoplasms, acute promyelocytic leukemia is extremely rare. Herein, we present a case of a promyelocytic blast crisis of post-ET myelofibrosis with associated life-threatening splanchnic vein thrombosis. This case suggests that inflammation plays a key role in thrombotic events and fibrotic/leukemic transformation in ET patients, regardless the molecular landscape.

## 1 Introduction

Essential thrombocythemia (ET) is a Philadelphia-negative myeloproliferative neoplasm (MPN) characterized by sustained thrombocytosis in the peripheral blood and increased numbers of giant mature megakaryocytes with hyperlobulated nuclei in a normocellular bone marrow without fibrosis. Life expectancy in ET is only mildly compromised and the main causes of disease-related death are thrombotic complications and fibrotic and/or leukemic transformation. Thrombotic complication occurs in 10–20% of ET patients. Thrombosis data from 1019 patients with ET considering three risk parameters (history of thrombosis, *JAK2/MPL* mutations, and advanced age) grouped ET patients into four risk categories: very low risk, low-risk, intermediate risk, and high risk group [1]. Patients with ET might develop bone marrow (BM) fibrosis, evolving into so-called WHO post-essential thrombocythemia myelofibrosis (MF), with median time to transformation of 7–20 years from ET diagnosis. The progression to myelofibrosis has a prevalence of 2.8% (10-year risk of 3.9%, 20-year risk of 19.9%) in ET patients [2]. Furthermore, leukemic progression occurs with less than 1% risk in the first decade of disease and 2% in 15 years in ET patients and reported risk factors for leukemic transformation in these patients have included anemia, extreme thrombocytosis, leukocytosis, presence of *TP53* or *EZH2* mutations and older age [3,4]. According to recent data from a large collaborative study of blast phase MPN on 410 patients, the most frequent types of blast crisis are acute myeloid leukemia (AML) with myelodysplasia-related changes and AML-not otherwise specified M7 [5]. No case of acute promyelocytic leukemia (APL) was reported in bone marrow examination morphology reports of this study, proving that transformation of MPN into APL is a rare, if not anecdotal event. Few cases on transformation of MPN to APL were reported in literature, and in majority of those the driver mutation status was unknown [6].

We report a promyelocytic blast crisis of *CALR* positive post ET-MF patient with associated splanchnic vein thrombosis (SVT). This case combines several extremely rare events: SVT in a fibrotic transformation of *CALR* mutated ET, and APL as MPN blast phase; unfortunately, COVID-19 infection nullified all our therapeutic achievements.

## 2 Case presentation

**Fig. 1:**
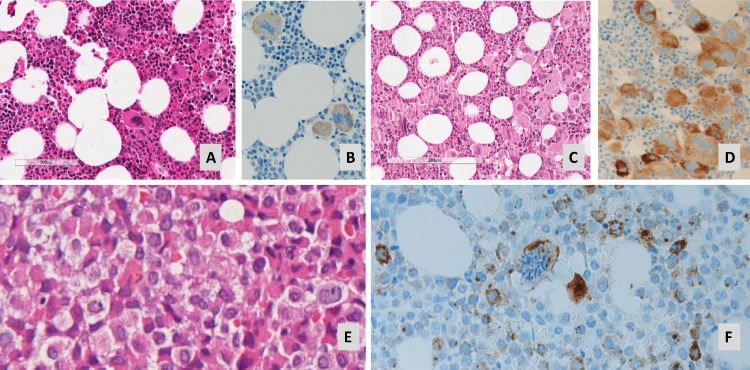
Evolution of bone marrow histology from 1994 to 2019. In 1994, MPN in chronic phase with ET morphology: normocellular bone marrow with enlarged megakaryocytes with hyperlobulated nuclei (A), reactive for CALR immunostaining (B). In 2016, progression to fibrotic phase as post-ET Myelofibrosis: hypercellular bone marrow with dense clusters of atypical megakaryocytes (C), reactive for CARL immunostaining (D). In 2019, APL-blast crisis with hypergranulated promyelocytes (E), staining positive for CALR together with a megakaryocyte (F)**.**

**Fig. 2:**
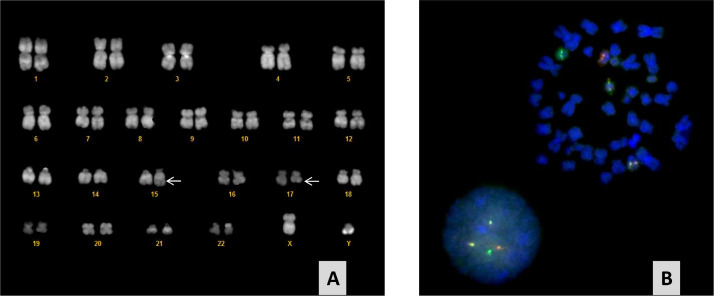
Conventional cytogenetic (A) and fluorescence in situ (B) at time of leukemic evolution. (A) G-banded karyotype revealed a reciprocal translocation between the long arms of chromosomes 15 and 17 at 15q22 and 17q21 [t(15;17)] in 20% of metaphases analyzed. An additional cytogenetic abnormality in chromosome 9 was found [del(9)(q21q32)]. (B) Interphase FISH demonstrated a fusion signal pattern of chromosome 15 and 17 in the patient. The PML gene in the chromosome 15 is labelled red, the RARα gene in the chromosome 17 is labelled green and the PML-RARα is labelled yellow.

A 55 years-old male suffering from long lasting chronic non erosive seronegative arthritis without health impairments was diagnosed with ET in 1994 (Fig. 1a) and began treatment with aspirin and hydroxyurea (HU). The disease remained stable for 22 years until June 2016 when he presented with severe abdominal pain, fever and anorexia. Physical examination was significant for pallor and palpable hepatomegaly and splenomegaly of new-onset. Abdominal computed tomography (CT) revealed portal, superior mesenteric and splenic vein thrombosis, massive splenomegaly with splenic infarct of 13 cm, multiple renal and hepatic infarcts, and floating thrombus of the abdominal aorta. A complete blood count showed anemia (hemoglobin level 8.8 g/dl), thrombocytosis (platelet count 1340 × 10^9^/L), and leukocytosis (white blood cell count 20,390 × 10^9^/L) with neutrophilia and presence of circulating myelocytes, metamyelocytes, and blasts (2%, 3%, and 1%, respectively). Work-up for the patient’s hypercoagulability revealed no alterations and blood biochemistries revealed abnormal liver function tests (grade 4 according Common Terminology Criteria for Adverse Events) and altered indices of inflammation. Molecular testing resulted positive for type 1 *CALR* mutation (variant allele frequency, VAF 58%) in absence of other mutations (including ASXL1, TET2, TP53, BCOR, RUNX1, NRAS, KRAS, SRSF2, DNMT3A, SF3B1, U2AF1, IDH1/2). Histologic examination showed a hypercellular bone marrow with increased granulopoiesis and megakaryocitopoiesis and increase in reticulin fibrosis (MF grade 2, according to EUMNET consensus). Atypical megakaryocytes formed loose clusters. Revision of bone marrow biopsy at diagnosis confirmed the diagnosis of ET and the second biopsy was referred to a post-ET myelofibrosis, categorized as intermediate-2 risk (MYSEC-PM). (Fig. 1a-d) The patient was managed with warfarin and ruxolitinib (15 mg BID), experiencing a progressive improvement of spleen size, liver impairment, and constitutional symptoms. After 6 months, a CT scan confirmed the resolution of aortic thrombosis, hepatic ischemic area, and half-reduction of the splenic ischemic area. However, the patient reported episodes of melena and hematemesis and several endoscopic ligations for varices were performed. On September 2018, another abdomen CT showed no recanalization of SVT. On March 2019, he presented with two-week history of worsening fatigue, weight loss and petechiae. Investigations revealed pancytopenia (total white cell count 3.37 × 10^9^/L, hemoglobin 8.5 g/dl, platelet count 10 × 10^9^/L), elevated lactate dehydrogenase, and no evidence of coagulopathy. The peripheral blood (PB) smear showed 12% blasts with cytoplasmic granules. Flow cytometry analysis of peripheral blood revealed a population of blast cells according for 30% of total cellularity. Conventional cytogenetic and fluorescence in situ (FISH) revealed the presence of t(15;17) (q24;q21) [14]/46, idem, del(9)(q21q32)[3] in 20% of metaphases of analyzed cells (Figs. 1*e* and 2**)**. Molecular studies were positive for PML/RARα and showed the persistence of *CALR* mutation type 1 (VAF 53%) in absence of other. Bone marrow biopsy exhibited diffuse sheets of immature myeloid cells and granulated promyelocytes with MPO+, CD15-, CD117-, CD34-, CD687PGM1-, and fibrosis grade 2, confirming the leukemic evolution. Bone marrow and peripheral blood examination, flow cytometry immunophenotyping, conventional cytogenetic and FISH yielded to the diagnosis of acute promyelocytic leukemia (APL). Immunohistochemistry was performed on serial bone marrow sections from 1994 to 2019 with a commercial antibody for immunodetection of all CALR mutations in ET and PMF (Anti-CALRmut, CAL2, Dianova Int.): megakaryocytes were positive in all specimens with promyelocytes and immature myeloid precursors staining positive in APL specimen (Fig. 1b-d-f). The patient was immediately started on all-trans retinoic acid (ATRA)/ arsenic trioxide (ATO) therapy for clinically intermediate-risk APL, according to Sanz. The patient tolerated well the ATO/ATRA regimen, with achievement of molecular complete response (CR) after consolidation. Moreover, at last control on December 2019, the BM showed reversion to the chronic phase MPN with grade 2 fibrosis. Platelets were increased, the *CALR* mutation persisted (VAF 44%), and SVT was stable. Therefore, he started again warfarin and HU. The patients remained APL-free for 9 months, but, unfortunately, died of COVID-19 pneumonia on February 2020.

## 3 Discussion and conclusion

A quarter of patients with ET carries as driver a mutation in calreticulin (*CALR)* gene. More than 80% of MPN patients with mutant *CALR* harbor 1 of 2 mutation variants: type 1, a 52-bp deletion (p.L367fs*46), or type 2, a 5-bp TTGTC insertion (p.K385fs*47) [7,8]. Several working groups have evaluated the role of driver mutations on the risk of thrombotic events, fibrosis transformation, and leukemic evolution. Overall survival and thrombosis-free survival data in 240 Mayo Clinic patients with ET were stratified by the presence of *JAK2 V617F* (*n* = 159), type 1 *CALR* (*n* = 46) or type 2 *CALR* (*n* = 35) mutation [9]. Among the two groups of patients *CALR* mutated, no differences in hemoglobin level, leukocyte count and IPSET score were reported. Moreover, there was no difference in overall survival between patients harboring *CALR* or *JAK2 V617F*, but thrombosis-free survival was significantly better in the former. According to Pietra et al., ET patients with *JAK2 V617F* or type 1-like *CALR* mutation were older, with lower platelet count, and higher incidence of thrombosis at diagnosis compared to ET patients carrying type 2-like *CALR* mutation. Furthermore, the reported 10-year cumulative incidence of thrombotic events was 14.9%, lower than in *JAK2* mutated patients (20.6%) [10]. These data reinforced the idea that the presence of *JAK2 V617F* predicts the development of thrombosis in MPN, and the unclear role of platelet count as predictive factor of thrombosis in MPN patients. Atypical thrombosis as SVT is a marker of aggressive disease biology and an independent risk factors for inferior survival in ET patient, associated also with severe organ damage [11]. Besides an overt or underlying MPN could be found in a large part of patients with non-cirrhotic SVT, only the 1.3% of these MPN harbored *CALR* mutation. Our case suggests that a major contribution to SVT formation in MPN patients could be from the chronic inflammation associated to MPN, accompanied by overt inflammatory cytokine production, bone marrow fibrosis, osteosclerosis and neoangiogenesis. As a matter of fact, in our patient SVT occurred with systemic symptoms considerable as an overlap between systemic inflammatory response symptoms (SIRS) and MPN including fever, loss of weight, night sweats, and blood count alterations. Moreover, concurrent to the symptoms and the thrombotic event, a diagnosis of fibrosis evolution was made. ET patients with type 1-like mutation were mainly associated with a significantly higher risk of myelofibrotic transformation; the reported 15-year cumulative incidence of MF was 26.2% in type 1-like *CALR* ET patients higher than type 2-like *CALR* ET and *JAK2 V617F* ET patients (5.4% and 6.9%, respectively). [10]

Approximately 5–10% of MPN patients experienced transformation to acute myeloid leukemia (AML). Concerning ET patients, leukemic transformation is considered a relatively uncommon event and published studies reported varied rates of AML evolution, from less than 1% to almost 10%. Gangat et al. identified age, anemia, and extreme thrombocytosis (>1000 × 109 L) as independent risk factors for leukemic evolution in this patients [3]. In addition to these, other reported risk factors were leukocytosis, and sequence variants/mutations involving *TP53* and *EZH2* [12]. A recent Mayo-AGIMM retrospective study of 410 patients from two separate cohorts is the largest study on blast-phase MPN published until now, but no APL were reported [5]. Moreover in literature, promyelocytic blast crisis of chronic myeloid leukemia, a Philadelphia-positive myeloproliferative disorder, are described in limited number of cases, even during treatment with tyrosine kinase inhibitor. [13]

At best of our knowledge, this report presents for the first time a case of APL as blast-phase MPN, arose from a type 1 *CALR* mutated ET. Using immunohistochemistry with CAL2 antibody, we observed that during the chronic phase, mainly the atypical megakaryocytes stained positive, but in the blastic phase also APL promyelocytes were clearly reactive for mutated *CALR*. Previous studies have shown that CAL2 immunohistochemistry in bone marrow is a specific, sensitive, rapid, simple and low-cost method for the detection of *CALR* mutations in ET and PMF [14], [15], [16]. In these studies the pattern of staining was limited to megakaryocytes and to rare myeloid precursors, in line with the emergence of *CALR* mutation in early multipotent progenitors. Similarly, our findings suggest that APL clone derived from MPN cells as a second genetic event in committed cells belonging to the *CALR* mutated MPN. The association between APL and MPN was thought to be due to de novo APL, APL secondary to prolonged cytoreductive therapy, and promyelocytic blast phase of MPN. The presence of CALR mutation in promyelocytic blast excluded the de novo etiology, as a not complex kayotype propended to exclude the therapy related AML.

Moreover, the management of the APL with ATO/ATRA treatment resulted in the achievement of a complete remission with the eradication of PML/RARA mutated clone followed by re-expansion of the clone harboring only *CALR* mutation, and with a morphologically return to post-ET myelofibrosis.

## 4 Compliance with ethical standards

### 4.1 Authorship and conflict-of-interest statements

All authors declare no conflict of interest regarding matters pertinent to the current manuscript. All authors have reviewed and approved the manuscript.

## Declaration of Competing Interest

The authors declare that they have no conflict of interest.

## References

[1] Barbui T., Vannucchi A.M., Buxhofer-Ausch V. (2015). Practice-relevant revision of IPSET-thrombosis based on 1019 patients with WHO-defined essential thrombocythemia. Blood Cancer J.

[2] Passamonti F., Rumi E., Arcaini L. (2008). Prognostic factors for thrombosis, myelofibrosis, and leukemia in essential thrombocythemia: a study of 605 patients. Haematologica.

[3] Gangat N., Wolanskyj A.P., McClure R.F. (2007). Risk stratification for survival and leukemic transformation in essential thrombocythemia: a single institutional study of 605 patients. Leukemia.

[4] Chim C.-.S., Kwong Y.-.L., Lie A.K.-.W. (2005). Long-term outcome of 231 patients with essential thrombocythemia. Arch Intern Med.

[5] Tefferi A., Mudireddy M., Mannelli F. (2018). Blast phase myeloproliferative neoplasm: mayo-AGIMM study of 410 patients from two separate cohorts. Leukemia.

[6] Mamorska-Dyga A., Wu J., Khattar P. (2016). Acute promyelocytic leukemia co-existing with JAK2 V617F positive myeloproliferative neoplasm: a case report. Stem Cell Investig.

[7] Nangalia J., Massie C.E., Baxter E.J. (2013). Somatic CALR mutations in myeloproliferative neoplasms with nonmutated JAK2. N Engl J Med.

[8] Klampfl T., Gisslinger H., Harutyunyan A.S. (2013). Somatic mutations of calreticulin in myeloproliferative neoplasms. N Engl J Med.

[9] Tefferi A., Wassie E.A., Guglielmelli P. (2014). Type 1 versus Type 2 calreticulin mutations in essential thrombocythemia: a collaborative study of 1027 patients. Am J Hematol.

[10] Pietra D., Rumi E., Ferretti V.V. (2016). Differential clinical effects of different mutation subtypes in CALR-mutant myeloproliferative neoplasms. Leukemia.

[11] Gangat N., Wolanskyj A.P., Tefferi A. (2006). Abdominal vein thrombosis in essential thrombocythemia: prevalence, clinical correlates, and prognostic implications. Eur J Haematol.

[12] Tefferi A., Lasho T.L., Finke C.M. (2016). Targeted deep sequencing in primary myelofibrosis. Blood Adv.

[13] Parsi M., Budak-alpdogan T. Promyelocytic blast crisis of chronic myeloid leukemia in a patient undergoing therapy with a tyrosine kinase inhibitor. 2020;12(3):3–7. doi:10.7759/cureus.7217. 10.7759/cureus.7217 PMC7141802 32274275

[14] Mózes R., Gángó A., Sulák A. (2019). Calreticulin mutation specific CAL2 immunohistochemistry accurately identifies rare calreticulin mutations in myeloproliferative neoplasms. Pathology.

[15] Andrici J., Farzin M., Clarkson A. (2016). Mutation specific immunohistochemistry is highly specific for the presence of calreticulin mutations in myeloproliferative neoplasms. Pathology.

[16] Stein H., Bob R., Dürkop H. (2016). A new monoclonal antibody (CAL2) detects CALRETICULIN mutations in formalin-fixed and paraffin-embedded bone marrow biopsies. Leukemia.

